# A multicenter evaluation of pediatric emergency department injury visits during the COVID-19 pandemic

**DOI:** 10.1186/s40621-023-00476-z

**Published:** 2023-12-13

**Authors:** Holly R. Hanson, Margaret Formica, Danielle Laraque-Arena, Mark R. Zonfrillo, Puja Desai, Joseph O. O’Neil, Purnima Unni, Estell Lenita Johnson, Patricia Cobb, Maneesha Agarwal, Kristen Beckworth, Stephanie Schroter, Stephen Strotmeyer, Katie A. Donnelly, Leah K. Middelberg, Amber M. Morse, James Dodington, Richard F. Latuska, Brit Anderson, Karla A. Lawson, Michael Valente, Michael N. Levas, Andrew Waititu Kiragu, Kathy Monroe, Stephanie M. Ruest, Lois K. Lee, Tanya Charyk Stewart, Megan M. Attridge, Maya Haasz, Mubeen Jafri, Alicia McIntire, Steven C. Rogers, Neil G. Uspal, Ashley Blanchard, Max D. Hazeltine, Teresa Riech, Charles Jennissen, Lynn Model, Quinney Fu, Lindsay D. Clukies, David Juang, Michelle T. Ruda, Jose M. Prince, Stephanie Chao, Brian K. Yorkgitis, Wendy J. Pomerantz

**Affiliations:** 1 Department of Pediatrics, Monroe Carell Jr. Children’s Hospital at Vanderbilt, Nashville, TN USA; 2 Department of Public Health and Preventive Medicine, SUNY Upstate Medical University, Syracuse, NY USA; 3 New York Academy of Medicine, New York, New York USA; 4 Clinical Epidemiology and Pediatrics, Mailman School of Public Health and Vagelos College of Physicians & Surgeons, Columbia University, Syracuse, NY USA; 5 Department of Emergency Medicine, Hasbro Children’s Hospital and Alpert Medical School of Brown University, Providence, RI USA; 6 Department of Pediatrics, Hasbro Children’s Hospital and Alpert Medical School of Brown University, Providence, RI USA; 7 Department of Pediatrics, St. Louis Children’s Hospital and Washington University School of Medicine, St. Louis, MO USA; 8 Department of Pediatrics, Indiana University, Indianapolis, IN USA; 9 Department of Pediatric Trauma, Monroe Carell Jr. Children’s Hospital at Vanderbilt, Nashville, TN USA; 10 Department of Epidemiology, School of Public Health, Injury Free Coalition for Kids, Columbia University, New York City, NY USA; 11 Division of Emergency Medicine, Cincinnati Children’s Hospital Medical Center, Cincinnati, OH USA; 12 Department of Pediatrics and Emergency Medicine, Emory University School of Medicine, Children’s Healthcare of Atlanta, Atlanta, GA USA; 13 Center for Childhood Injury Prevention, Texas Children’s Hospital, Houston, TX USA; 14 Department of Pediatric Emergency Medicine, University of California, Rady Children’s Hospital San Diego, San Diego, CA USA; 15 Department of Pediatric General and Thoracic Surgery, UPMC Children’s Hospital of Pittsburgh, Pittsburgh, PA USA; 16 Allegheny County Health Department, Pittsburgh, PA USA; 17 Division of Emergency Medicine, Children’s National Hospital, Washington, DC USA; 18 Division of Emergency Medicine, Department of Pediatrics, Nationwide Children’s Hospital, Columbus, OH USA; 19 Division of Pediatric Emergency Medicine, University of Arkansas for Medical Sciences, Arkansas Children’s Hospital, Little Rock, AR USA; 20 Yale School of Medicine, New Haven, CT USA; 21 Division of Emergency Medicine, Children’s Hospital of Philadelphia, Philadelphia, PA USA; 22 Department of Pediatric Emergency Medicine, Norton Children’s Hospital, Louisville, KY USA; 23 Trauma and Injury Research Center, Dell Children’s Medical Center of Central Texas, Austin, TX USA; 24 Department of Pediatric Emergency Medicine, Children’s Health Orange County, Orange, CA USA; 25 Department of Pediatric Emergency Medicine, Medical College of Wisconsin, Milwaukee, WI USA; 26 Department of Pediatrics, University of Minnesota and Children’s Minnesota, Minneapolis, MN USA; 27 Division of Pediatric Emergency Medicine, University of Alabama at Birmingham, Birmingham, AL USA; 28 Division of Emergency Medicine, Boston Children’s Hospital, Boston, MA USA; 29 Department of Paediatrics, University of Western Ontario and London Health Sciences Centre, London, ON Canada; 30 Division of Emergency Medicine, Ann and Robert H. Lurie Children’s Hospital of Chicago, Chicago, IL USA; 31 Department of Pediatrics, University of Colorado School of Medicine, Aurora, CO USA; 32 Department of Pediatric Surgery, Oregon Health and Science University, Portland, OR USA; 33 Department of Pediatric Surgery, Randall Children’s Hospital at Emanuel Legacy, Portland, OR USA; 34 Department of Emergency Medicine, University of Connecticut School of Medicine, Connecticut Children’s Hospital, Hartford, CT USA; 35 Division of Emergency Medicine, University of Washington, Seattle Children’s Hospital, Seattle, WA USA; 36 Department of Emergency Medicine, Columbia University Medical Center, New York City, NY USA; 37 Department of Surgery, UMass Chan Medical School, Worcester, MA USA; 38 Department of Pediatric Emergency Medicine, OSF Saint Francis Medical Center, Peoria, IL USA; 39 Department of Pediatrics, Roy J. and Lucille A. Carter College of Medicine, University of Iowa, Iowa City, IA USA; 40 Department of Emergency Medicine, Roy J. and Lucille A. Carter College of Medicine, University of Iowa, Iowa City, IA USA; 41 Department of Pediatric Surgery, Maimonides Medical Center, Brooklyn, NY USA; 42 Division of Emergency Medicine, Phoenix Children’s Hospital, Phoenix, AZ USA; 43 Department of Pediatrics, Washington University in St. Louis School of Medicine, St. Louis Children’s Hospital, St. Louis, MO USA; 44 Department of Pediatric Surgery, Children’s Mercy Hospital, Kansas City, MO USA; 45 Department of Pediatrics, Children’s Memorial Hermann Hospital, UTHealth Houston, Houston, TX USA; 46 Department of Pediatric Surgery, Northwell Health, New Hyde Park, NY USA; 47 Division of Pediatric Surgery, Stanford School of Medicine, Palo Alto, CA USA; 48 Department of Surgery, University of FL College of Medicine – Jacksonville, Jacksonville, FL USA; 49 Division of Emergency Medicine, University of Cincinnati College of Medicine, Cincinnati Children’s Hospital Medical Center, Cincinnati, OH USA; 50 Cincinnati Children’s Hospital Medical Center, 3333 Burnet Ave, Cincinnati, OH 45229 USA

**Keywords:** Pediatrics, Injury prevention, Injuries, Emergency department, Pandemic, Disparities

## Abstract

**Background:**

Injuries, the leading cause of death in children 1–17 years old, are often preventable. Injury patterns are impacted by changes in the child’s environment, shifts in supervision, and caregiver stressors. The objective of this study was to evaluate the incidence and proportion of injuries, mechanisms, and severity seen in Pediatric Emergency Departments (PEDs) during the COVID-19 pandemic.

**Methods:**

This multicenter, cross-sectional study from January 2019 through December 2020 examined visits to 40 PEDs for children < 18 years old. Injury was defined by at least one International Classification of Disease-10th revision (ICD-10) code for bodily injury (S00–T78). The main study outcomes were total and proportion of PED injury-related visits compared to all visits in March through December 2020 and to the same months in 2019. Weekly injury visits as a percentage of total PED visits were calculated for all weeks between January 2019 and December 2020.

**Results:**

The study included 741,418 PED visits for injuries pre-COVID-19 pandemic (2019) and during the COVID-19 pandemic (2020). Overall PED visits from all causes decreased 27.4% in March to December 2020 compared to the same time frame in 2019; however, the proportion of injury-related PED visits in 2020 increased by 37.7%. In 2020, injured children were younger (median age 6.31 years vs 7.31 in 2019), more commonly White (54% vs 50%, *p* < 0.001), non-Hispanic (72% vs 69%, *p* < 0.001) and had private insurance (35% vs 32%, *p* < 0.001). Injury hospitalizations increased 2.2% (*p* < 0.001) and deaths increased 0.03% (*p* < 0.001) in 2020 compared to 2019. Mean injury severity score increased (2.2 to 2.4, *p* < 0.001) between 2019 and 2020. Injuries declined for struck by/against (− 4.9%) and overexertion (− 1.2%) mechanisms. Injuries proportionally increased for pedal cycles (2.8%), cut/pierce (1.5%), motor vehicle occupant (0.9%), other transportation (0.6%), fire/burn (0.5%) and firearms (0.3%) compared to all injuries in 2020 versus 2019.

**Conclusions:**

The proportion of PED injury-related visits in March through December 2020 increased compared to the same months in 2019. Racial and payor differences were noted. Mechanisms of injury seen in the PED during 2020 changed compared to 2019, and this can inform injury prevention initiatives.

## Introduction

Injuries are the leading cause of death and disability in children 1–17 years of age in the United States (US) (Centers for Disease Control and Prevention Injury Center of Injury Prevention and Control 2022). Infectious diseases and cancer, once primary contributors to pediatric deaths, have decreased with advancements in medical science, while injuries have become the leading cause of death (Cunningham et al. 2018). The environment, an interaction of physical, social, economic, cultural, and demographic components, affects a child’s risk of injury (Peek-Asa and Zwerling 2003). National disasters, global infectious disease burden, and economic crises are examples of environmental changes that have impacted pediatric injuries (Huang et al. 2011). The United States economic recession in 2007–2009 saw an increased in abusive head trauma in children (Huang et al. 2011; Wood et al. 2016). During this same time, disparities in traumatic injuries were seen as areas hit hardest with unemployment also had the highest hospital admission rates for trauma (Coughlin et al. 2018). During the Middle East Respiratory Syndrome (MERS) outbreak in June and July 2015, a study looking at ED utilization rates found that visits for pediatric injury increased by 4.1% compared to similar months in the two prior years (Paek et al. 2017). Studies have consistently shown that influencing these environmental factors are poverty level, access to needed goods and services, and personal and societal anxiety and fear (Drake and Rank 2009; Mack et al. 2018).

On March 13, 2020, the US declared a National Emergency due to the emergence of the novel coronavirus known as SARS-CoV-2 (or COVID-19) (Trump 2020). Within 1 month nearly all 50 states had enacted “shelter-in-place” orders requiring residents to stay within their homes. This resulted in a nationwide closure of schools, playgrounds, daycares, and all places of business deemed “non-essential”. A paradigm emerged where children were home for long periods of time with adult caregivers, who often were required to work full-time from home while also having to care for or provide schooling to children (Claudet et al. 2020). Studies assessing the first months of the pandemic demonstrated a profound decrease in pediatric emergency department (PED) visits (Finkelstein et al. 2021; Delaroche et al. 2021). Some hypothesized that increases in injuries would be demonstrated as a result of the conditions imposed by the pandemic, while others speculated a decrease (Gelder et al. 2020; Keays et al. 2020; Sutherland et al. 2020). The objective of this study was to examine changes in the epidemiology of pediatric injuries during the COVID-19 pandemic among children’s hospitals. We hypothesized that there would be a relative increase in the proportion of injury-related PED visits, with changing patterns of injury mechanism, due to behavior changes during the pandemic. Understanding how widespread environmental changes can influence injury patterns may be useful for informing further injury prevention efforts.

## Methods

### Study design and data collection

**Fig. 1:**
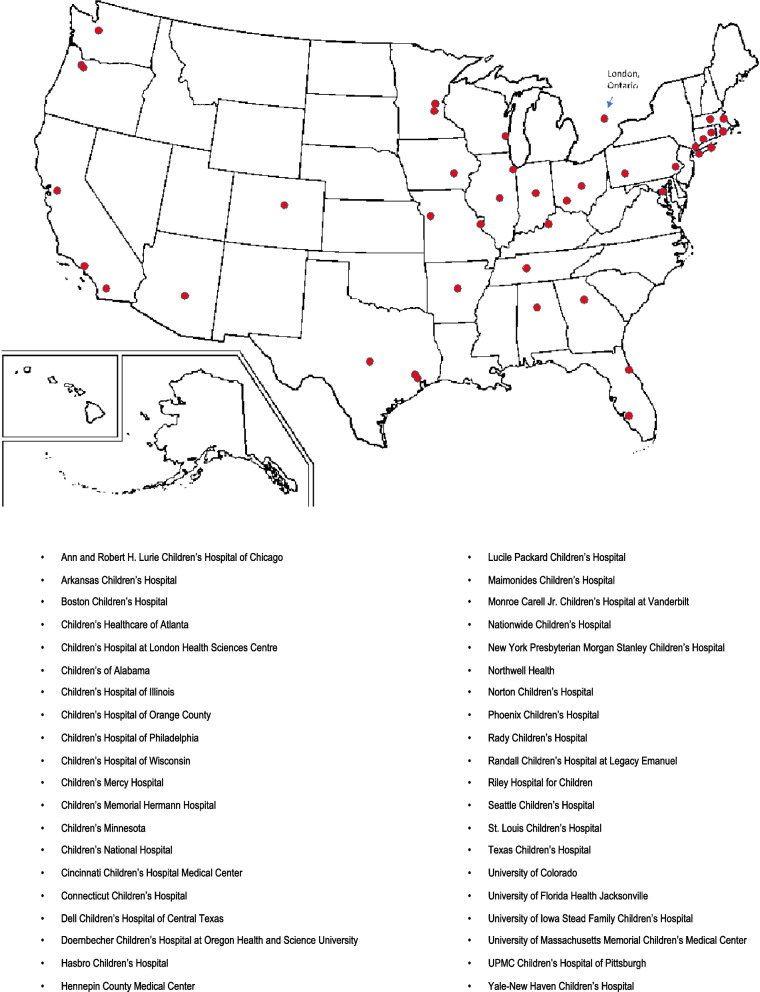
Map of participating sites

We conducted a multicenter, cross-sectional study of 40 hospital PEDs across the USA and Canada (Fig. 1). This work was supported by the Injury Free Coalition for Kids® (IFCK®)*,* a hospital-based, community-oriented organization led by pediatric physicians, surgeons, and injury prevention specialists. The purpose of the IFCK® is to reduce all injuries to children. The study team comprised 36 hospitals from IFCK® sites and 4 from additional institutions. Study participation was offered to each IFCK® site and was optional. We included all visits to a PED for children less than 18 years of age presenting between January 1, 2019, and December 31, 2020, with an injury. Injury was defined by at least one International Classification of Disease-10th revision (ICD-10) code for bodily injury (S00–T78). We excluded any visit for the same injury within 7 days and those occurring as a complication of surgical and/or medical care (ICD-10 codes T80-88, Y65.8). The study was approved by the Institutional Review Boards at all sites.

### Study protocol

Eligible subjects were identified retrospectively using instructions provided to each enrollment site in a standardized manual of operations (MOO) and data dictionary. All study variables were defined a priori. Each investigator received standardized training, and all data were abstracted directly from the electronic medical record at each institution and uploaded into a REDCap database housed at Cincinnati Children’s Hospital Medical Center, the study’s data coordinating site (Harris et al. 2009). Data were assessed for completeness and accuracy. Any noted discrepancies were reviewed with primary sites. Data sources included PED records, hospital admission records, trauma registries, and ICD-10 billing codes.

### Patient characteristics

Demographic characteristics collected for each PED visit included age, sex, race, ethnicity, payor, hospital, region of the country, and home zip code. Race and ethnicity designations were typically based on self-report or hospital registration assignment, per institutional practice. Region was categorized using the US Census designations: Northeast (CT, DC, MA, NY, PA, RI), Midwest (IA, IL, IN, MO, MN, NE, OH), South (Al, AR, FL, GA, KY, TN, TX), West (AZ, CA, CO, OR, WA), and Canada. Injury-related variables included date/time of PED visit and hospital discharge, emergency severity index (ESI) code (range 1–5 with 1 assigned to the most urgent patients), trauma activation (yes/no), PED disposition, admission site (inpatient floor, intensive care unit, operating room, burn unit, psychiatric unit), and outcome (lived/died). PED disposition includes death in the PED, and conversely, outcome (lived/died) includes death at any point in the hospital encounter including after hospital admission. The first twenty ICD-10 codes that corresponded to bodily injury were collected. The first three ICD-10 codes for mechanism and intent of injury (V00–X58 = unintentional, X71–X83 = intentional self-harm, X92–Y09 = assault, Y21–Y33 = undetermined intent, Y35–Y38 = legal intervention) were recorded. For each hospital, the total number of PED visits seen for all causes by week was obtained.

### Derived data

Injury severity scores (ISSs) were derived for each visit. The abbreviated injury scale (AIS) is anatomically based and consensus derived. It is the global lexicon of choice for injury severity determination and is used to generate an ISS (Association for the Advancement of Automotive Medicine (AAAM) 2008). The AIS documents each injury sustained by body region and severity on a 1 (minor) to 6 (maximal) scale. AIS scores were derived electronically using a validated map from all ICD-10-CM injury diagnosis codes for each visit (Loftis et al. 2005; Glerum and Zonfrillo 2019). ISSs were calculated by summing the squares of the AIS scores of the three most severely injured body regions with a possible ISS range of 0–75 (Association for the Advancement of Automotive Medicine (AAAM) 2008). If an injury is unsurvivable, denoted by an AIS of 6, the ISS is automatically assigned a value of 75. Additionally, an ISS of 0 indicates an injury where specific body region damage was absent, for example, poisoning and drownings.

A deprivation index was added to each patient encounter. The index, available for each US zip code, is formulated based on a principal components analysis of six American Community Survey measures from US census data (Brokamp et al. 2018, 2019). Those measures include the fraction of the population with income below poverty level, those over 25 year of age with a high school diploma (or equivalent), those with no health insurance, those receiving public assistance income or food stamps, the fraction of vacant homes, and the median household income in the past 12 months (Brokamp et al. 2018). The index provides a number 0 to 1 with 1 representing the most socioeconomically disadvantaged areas.

### Data analysis

Data analysis was performed at the State University of New York Upstate Medical University. For comparisons of injuries and PED visits before (2019) and during the pandemic (2020), visits were limited to those occurring beginning week 12 of each calendar year, the first full week of the pandemic in 2020. We included visits occurring beginning March 17th in 2019 and March 15th in 2020. Weekly injury visits as a percentage of total PED visits were calculated for all weeks between January 2019 and December 2020 by dividing the total number of injury PED visits each week by the total number of PED visits each week and multiplying by one hundred. Three sites were unable to provide data on total PED visits by week and were excluded from this calculation. Descriptive statistics were calculated for demographic and clinical characteristics and were compared between 2019 and 2020 using Chi-square and *t*-tests, as appropriate, with *p* values < 0.05 deemed statistically significant. Race and ethnicity information were missing in 8% and 8.7% of cases, respectively, and were similar in 2019 and 2020. Descriptive statistics were used to characterize injury data overall and by week and year. The Centers for Disease Control and Prevention (CDC) National Center for Health Statistics Injury Diagnosis Matrices and External Cause-of-Injury (E-Code) Matrices were used to classify nature, mechanism, and intent of injury (Hedegaard et al. 2019, 2020). All statistical analyses were conducted using SAS® (version 9.4, SAS Institute, Inc., Cary, NC).

## Results

**Fig. 2:**
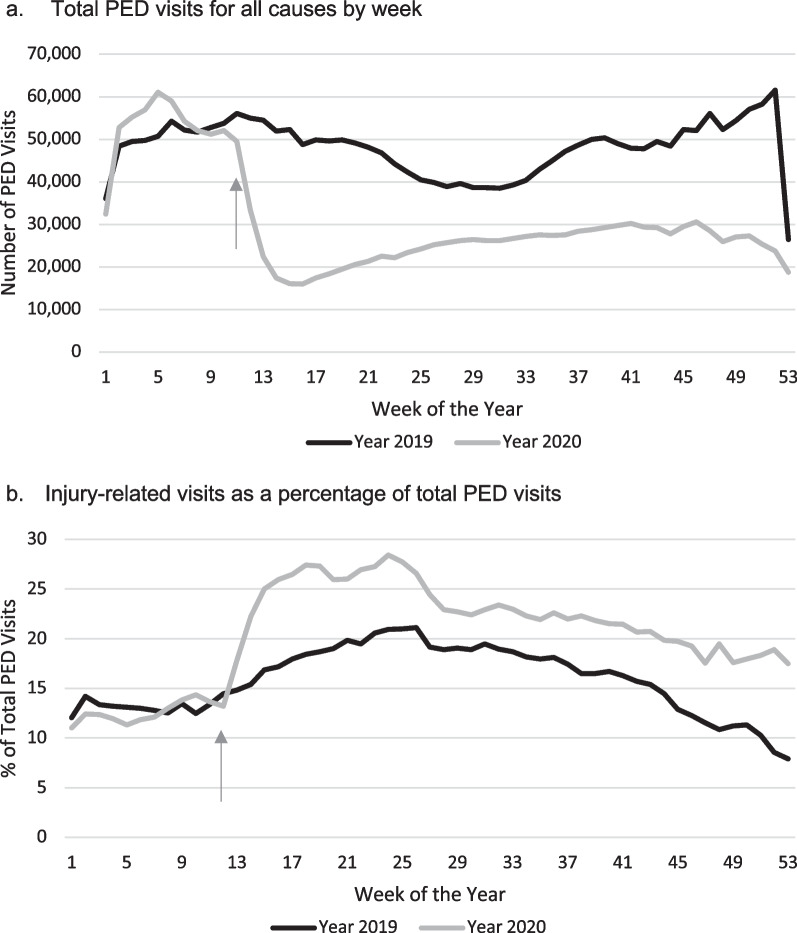
Weekly trends in Pediatric Emergency Department (PED) visits. **a** Total PED visits for all causes by week. **b** Injury visits as a percentage of total PED visits. *Arrow denotes week 12 when the USA declared a National Emergency

There were 4,181,297 total PED visits for all causes (injury and non-injury) from January 1, 2019, through December 31, 2020. This number represents data from 37 out of the 40 sites. Three sites were unable to contribute data for all causes of PED visits due to technical difficulty in data extraction from the site-specific electronic medical record. There was a 27.4% decrease in visits for all causes from March through December 2020 compared to the same months in 2019 (Fig. 2a).

There were 741,418 injury-related PED visits at 40 participating hospitals from January 1, 2019, through December 31, 2020. Focusing on injuries that occurred during the pandemic months, between March and December of 2019 and 2020 there were 589,083 injury-related PED visits (341,242 in 2019; 247,841 in 2020). There were 93,401 less visits for injuries during March to December 2020 compared to March to December 2019. Compared to the 27.4% decrease in ED visits for all causes, there was a 37.7% increase in the proportion of injury-related visits from March through December 2020 compared to the same months in 2019 (Fig. 2b).

Looking at data from March to December, children who presented to the PED for injuries during the COVID-19 pandemic in 2020 were younger (median age 6.3 years vs 7.3 in 2019), more frequently White, and privately insured (Table 1). The median deprivation index for patients evaluated for injuries decreased from 0.4 in 2019 to 0.3 in 2020 (*p* < 0.001). During the COVID-19 pandemic, there was a higher percentage of trauma activations (+ 1.5%) and patients triaged at ESI levels 1 and 2 (+ 2.5%). A higher percentage of patients presented to the PED in the evening or overnight (+ 2.5%).

**Table 1:** Demographic and PED injury-related visit characteristics before and during the COVID-19 pandemic

Characteristic	March–December 2019 (*n* = 341,242)	March–December 2020 (*n* = 247,841)	*p* value*
*Age, No.* (%), years			< 0.001
< 1	20,717 (6.1)	15,888 (6.4)	
1–4	105,877 (31.0)	87,418 (35.3)	
5–9	86,769 (25.4)	52,058 (25.0)	
10–14	84,736 (24.8)	52,901 (21.3)	
15–18	43,143 (12.6)	29,576 (11.9)	
*Sex, No.* (%)			< 0.001
Male	194,171 (56.9)	138,592 (55.9)	
Female	145,102 (42.5)	108,500 (43.8)	
Unknown	1,969 (0.6)	749 (0.3)	
*Race, No.* (%)^a^			< 0.001
White	169,694 (49.7)	133,135 (53.7)	
Black	85,383 (25.0)	57,775 (23.3)	
AI/AN	1,177 (0.4)	888 (0.4)	
Asian	9,252 (2.7)	6,504 (2.6)	
Multiple	8,641 (2.5)	7,027 (2.8)	
Other	37,350 (11.0)	24,619 (9.9)	
Unknown	29,745 (8.7)	17,893 (7.3)	
*Ethnicity, No.* (%)^a^			< 0.001
Hispanic	74,271 (21.8)	50,385 (20.3)	
Non-Hispanic	235,485 (69.0)	177,476 (71.6)	
Unknown	31,486 (9.2)	19,980 (8.1)	
*Payor, No.* (%)			< 0.001
Public	176,606 (51.7)	120,583 (48.7)	
Private	108,976 (31.9)	86,028 (34.7)	
Military	4,083 (1.2)	3,200 (1.3)	
Self	13,142 (3.9)	9,920 (4.0)	
Canadian	8,594 (2.5)	5,457 (2.2)	
International	218 (0.1)	100 (0.0)	
Other/unknown	29,623 (8.7)	22,553 (9.1)	
*Deprivation Index* (0–1)			
Median (IQR)	0.4 (0.3–0.4)	0.3 (0.3–0.4)	< 0.001
*Triage ESI Code, No.* (%)			< 0.001
1	3,635 (1.1)	3,434 (1.4)	
2	53,545 (15.7)	44,365 (17.9)	
3	122,556 (35.9)	92,142 (37.2)	
4	140,686 (41.2)	95,281 (38.4)	
5	14,340 (4.2)	8,177 (3.3)	
Unknown	6,480 (1.9)	4,442 (1.8)	
*Trauma activation, No.* (%)			< 0.001
Yes	23,562 (6.9)	20,706 (8.4)	
No	238,841 (70.0)	173,335 (69.9)	
Unknown	78,839 (23.1)	53,800 (21.7)	
*Time of day PED visit, No.* (%)^b^			< 0.001
8:00–15:59	113,554 (35.0)	75,897 (32.5)	
1600–23:59	169,740 (52.4)	125,644 (53.7)	
00:00–07:59	40,820 (12.6)	32,323 (13.8)	
*Day of PED visit, No.* (%)			< 0.001
Sunday	50,649 (14.8)	38,070 (15.4)	
Monday	50,686 (14.9)	36,498 (14.7)	
Tuesday	48,358 (14.2)	34,491 (13.9)	
Wednesday	47,360 (13.9)	33,929 (13.7)	
Thursday	46,933 (13.8)	34,047 (13.7)	
Friday	47,987 (14.0)	33,757 (13.6)	
Saturday	49,269 (14.4)	37,049 (15.0)	
Weekday	24,1324 (70.7)	172,722 (69.7)	
Weekend	99,918 (29.3)	75,119 (30.3)	
PED length of stay, hours, median (IQR)^c^	3 (2–4)	2 (2–4)	0.691
Hospital length of stay, hours, median (IQR)^c^	36 (20–72)	36 (20–75)	0.006
*Region, No.* (%)			< 0.001
Northeast	95,208 (27.9)	71,879 (29.0)	
Midwest	78,623 (23.1)	60,139 (24.3)	
South	102,463 (30.0)	76,721 (31.0)	
West	56,330 (16.5)	33,640 (13.5)	
Canada	8618 (2.5)	5462 (2.2)	

AI/AN, American Indian/Alaskan Native; PED, Pediatric Emergency Department; ESI, emergency severity index

*Where the *p* value is listed on the characteristic line, the *p* value is representative of a difference noted in the category

^a^Race and ethnicity numbers may have some overlap as these are not all mutually exclusive

^b^Missing data from one site (*n* = 557,978)

^c^Improbable values for PED length of stay (> 48 h) and hospital length of stay (> 180 days) were excluded. This included *n* = 1892 in the PED length of stay and *n* = 83 in the hospital length of stay

**Fig. 3:**
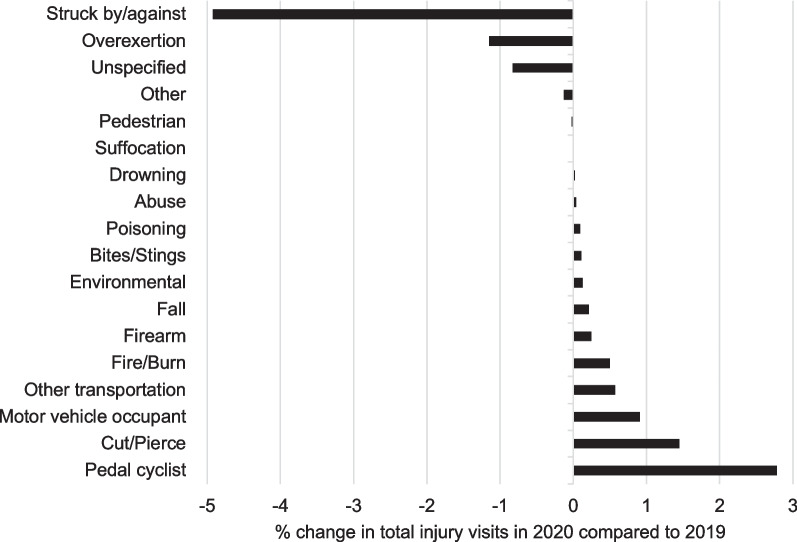
Percentage change in mechanisms of injury in 2020 versus 2019. Among *N* = 243,969 visits with classifiable external cause codes

A higher proportion of injuries presenting to PEDs during the pandemic resulted in admission to the hospital (+ 2.2%) and intensive care unit (+ 0.9%) and in a greater percentage of deaths (+ 0.03%) (Table 2). The mean ISS increased marginally in 2020 compared to 2019; however, greater percentages of injuries shifted into the “moderate” to “very severe” ISS categories in 2020. Only 46.2% (274,404) of all injury-related visits had a mechanism/intent documented. Of those documented, there were more intentional self-harm (+ 0.4%) and fewer assaults (− 1.0%) in 2020 (Table 2). The top two reported mechanisms both years were falls and struck by/against. There was a decrease in the percentage of injuries caused by struck by/against and overexertion mechanisms in 2020 compared to 2019 (Fig. 3). Pedal cyclist injuries demonstrated the largest change of any mechanism (+ 2.8%) from 2019 to 2020. Other injuries which increased included cut/pierce, motor vehicle occupant crash, other transportation crash (including all-terrain vehicle), and fire/burn, which each had a > 0.5% increase in the proportion of PED visits in 2020 (Fig. 3). Notably, the proportion of injuries that were the result of a firearm increased nearly twofold (0.35–0.60%) from 2019 to 2020, and in 2020, 127 more children were injured by firearms compared to 2019.

**Table 2:** Injury pattern characteristics before and during the COVID-19 pandemic

Characteristic	March–December 2019 (*n* = 341,242)	March–December 2020 (*n* = 247,841)	*p* value*
ISS, mean (SD)	2.2 (2.6)	2.4 (2.9)	< 0.001
*ISS categories, No.* (%)			< 0.001
0	96,729 (29.0)	67,673 (27.9)	
1–8 (Mild)	226,040 (67.8)	164,827 (68.1)	
9–15 (Moderate)	9089 (2.7)	8160 (3.4)	
16–24 (Severe)	872 (0.3)	865 (0.4)	
≥ 25 (Very severe)	485 (0.2)	486 (0.2)	
*Intent, No.* (%)^a^			< 0.001
Unintentional	152,587 (95.7)	108,716 (96.2)	
Intentional self-harm	1687 (1.1)	1744 (1.5)	
Assault	4630 (2.9)	2197 (1.9)	
Undetermined intent	392 (0.2)	345 (0.3)	
Legal intervention	82 (0.1)	24 (0.1)	
*PED disposition, No.* (%)			< 0.001
Discharge	298,190 (87.4)	210,777 (85.0)	
Admit	36,759 (10.8)	32,149 (13.0)	
Died	87 (0.0)	122 (0.1)	
Other^b^/unknown	6206 (1.8)	4793 (1.9)	
*Admitting unit, No.* (%)^c^			< 0.001
Ward	26,176 (71.2)	22,736 (70.7)	
Intensive care unit	4059 (11.0)	3831 (11.9)	
Operating room	3537 (9.6)	2679 (8.3)	
Psychiatry	966 (2.6)	833 (2.7)	
Burn	522 (1.5)	452 (1.4)	
Unknown	1499 (4.1)	1618 (5.0)	
*Status, No.* (%)			< 0.001
Lived	317,254 (93.0)	229,510 (92.6)	
Died	335 (0.1)	327 (0.1)	
Unknown	23,653 (6.9)	18,004 (7.3)	

PED, Pediatric Emergency Department; ISS, Injury Severity Score

*Where the *p* value is listed on the title line of the characteristic, the *p* value is representative of a difference noted in the category

^a^Among *n* = 272,404 with classifiable external cause codes

^b^“Other” includes transfer to another facility for psychiatric admission, transfer to another hospital, and left prior to formal discharge

^c^Among *n* = 68,908 patients admitted to hospital

When considering types of bodily injuries, a lower percentage of injuries presenting to the PED had superficial contusions (− 1.7%) and internal organ injuries (− 0.4%) in 2020 compared to 2019. However, there were increases in lacerations (+ 4.9%), fractures/dislocations (+ 1.5%), poisonings (+ 1.3%), retained foreign bodies (+ 1.2%), burns (+ 0.5%), crush/amputations (+ 0.07%), and vascular injuries (+ 0.04%).

## Discussion

During the first months of the COVID-19 pandemic, the world saw dramatic shifts in work, home, school, and recreational activities (Claudet et al. 2020). While visits to PEDs for all causes decreased across the country, we sought to evaluate the impact of this change on pediatric injuries specifically. We found that the number of PED visits for all causes decreased 27% across the first 9 months of the pandemic; however, the proportion of injury-related PED visits increased by 38%. A greater percentage of these injury-related visits resulted in hospital admissions and deaths. Mechanisms often due to recreational and sport activities (e.g., struck by/against and overexertion) decreased dramatically, whereas injuries from single-user activities (e.g., bicycle, all-terrain vehicles, horseback riding) increased. Proportions of firearm injuries, poisonings, and burns, which may have occurred at home, also increased.

Similar to prior studies, our data demonstrate a decrease in PED visits for all causes throughout the first 9 months of the pandemic (Finkelstein et al. 2021; Delaroche et al. 2021; Lowe et al. 2021; Romano et al. 2021). Specific to injuries, a study using data from an administrative database of children’s hospitals reported a 26.6% decrease in PED injury-related visits during the first year of the COVID-19 pandemic (Lowe et al. 2021). This study defined injury differently than our study. Our study examined all ED visits for any bodily injury using S and T ICD-10 codes, while the previous study identified injuries by mechanism. We found that among our 40 sites, 53.8% of the population was missing a billing code for mechanism. Thus, the previous study may have been an under-representation of all pediatric injuries.

The COVID-19 pandemic drew attention to US racial, ethnic, and socioeconomic disparities in infection rates, access to medical care, and vaccination status (Lowe et al. 2021; Romano et al. 2021; Kricorian and Turner 2021). Individuals with lower socioeconomic status, public insurance and those from marginalized populations were less likely to seek care in an ED during the pandemic (Lowe et al. 2021). This is in contrast to ED utilization prior to the pandemic where individuals with public insurance were more likely to utilize the ED for various reasons, such as less access to after-hours primary care (Sen et al. 2022; Chande et al. 1996). In our study, there was a decrease in the median deprivation index of those individuals who sought PED care in 2020, indicating less PED visits from socioeconomically disadvantaged areas. Additionally, we found that pediatric patients who were White and who had private insurance were a higher proportion of those presenting with an injury during the study pandemic year. Although reasons for this are not well understood, this may be related to the same reasons why those from marginalized populations had lower overall ED visits rates during the pandemic (Lowe et al. 2021).

Our data shows a higher proportion of PED injury-related visits were assigned ESI levels indicating more severe injuries and that a greater percentage were trauma activations during 2020 compared to the year prior. While the small difference in mean ISS between the 2 years represent minor injury and may not be clinically meaningful, we did find that a higher proportion of injuries shifted into the “moderate”-to-“very-severe” ISS categories. The triangulation of these elevated surrogates for injury severity, in addition to the 0.03% increase in the proportion of deaths, resulting in an additional 35 deaths in children, indicates that injuries were overall more severe and outcomes worse in our population during the pandemic. Our study population also demonstrated a 2.2% increase in the proportion of injuries requiring hospital admission and a nearly 1% increase in the percentage admitted to the intensive care unit during the COVID-19 pandemic. A similar pattern has been documented in a prior study (Wells et al. 2022). The significance of these increases is further highlighted when considering that hospitalizations decreased for all causes during the same timeframe (Pelletier et al. 2020).

We saw changes in the mechanisms of injury presenting to PEDs during the pandemic compared to the year prior. The proportion of injuries from being struck by/against and overexertion decreased in 2020. Sports-related injuries contribute to both mechanisms, and studies have shown a decrease in sports participation and sport-related injury visits to PEDs during the pandemic (Sabbagh et al. 2022; Post et al. 2022). Pedal cyclist saw the highest percentage increase in injuries in our population during the pandemic. Bicycle sales increased in 2020 and may be due to factors including more time doing single-user activities, an increasing desire for fitness, or a need for transportation with lesser perceived infection risk than some public options (Dowell and Hait 2021). Our findings are consistent with other studies reporting the same increase in cyclist injuries (Wells et al. 2022; Oudtshoorn et al. 2021).

In addition to these changes in injury mechanisms, we report additional increases in several other mechanism types including motor vehicle occupant crashes and firearm-related injuries. We found a nearly 1% increase in the proportion of injuries from motor vehicle occupant crashes. This same trend has been reported in other studies, including data from the National Highway Traffic Safety Administration showing a 6.8% increase in traffic fatalities for all ages in 2020 compared to 2019 (Chaudhari et al. 2022; Stewart 2020). Additionally, firearm injuries have gained increased national attention as they are now the number one cause of death in children over the age of 1 year (Lee et al. 2022). A multi-institutional study of nine Level 1 pediatric trauma centers found an 87% greater odds of injured pediatric patients presenting with a firearm injury during the pandemic compared to the year prior (Collings et al. 2022). Our study supports these findings with a nearly doubling of the percentage of injuries due to firearms seen in 2020 as compared to 2019. Prior studies have shown that firearm purchases increased during the pandemic and parents describe storing firearms in more accessible locations (Schleimer et al. 2021; Sokol et al. 2021). It is possible that children being home with more access to firearms may contribute to the increase. Regardless, the morbidity and mortality associated with this mechanism are great and thus must be a focus for policy and prevention efforts.

The intent of injury changed during the pandemic with increases in the proportion of injury-related PED visits that were categorized as intentional self-harm. These injuries are likely associated with depression and/or suicidal ideation (e.g., intention cutting) or suicide attempts. The effects of the COVID-19 pandemic on mental health have been staggering (Moreno et al. 2020; Hafstad and Augusti 2021). Experts in psychiatry warned early in the pandemic of the possible effects that social distancing, isolation, and fear might have on mental health and suicide rates (Gunnell et al. 2020; John et al. 2020). A renewed focus on mental health is imperative for children to begin to address and mitigate the effects of the pandemic. PEDs are often underequipped to address the increasing population of patients presenting for self-harm, which warrants further funding and investigation.

This study has a number of limitations. These data are retrospective and extracted from the electronic health records resulting in some missing information. We do not have information on disability or long-term outcomes. Injury mechanisms and intent were not consistently documented, or categorized as non-specific; therefore, we could not accurately classify all injury types. Information was also missing for some demographic characteristics, such as race and ethnicity. We searched for injuries based specifically on ICD-10 codes for injury type to provide a thorough review of all potential injuries, implemented robust quality assurance and control measures in the study protocol, and found that missing data were similar across the years, limiting bias. We also recognize that hospital resources and admitting units across institutions differ, which may have resulted in some misclassification. Finally, the data presented in this study are the representation of 40 hospitals, representing 25 states, across the USA and Canada, but are not population-based data. We recognize that there are several large databases that address pediatric injuries, including but not limited to the National Electronic Injury Surveillance System (NEISS), National Trauma Data Bank, and Web-based Injury Statistics Query and Reporting System. We chose to establish our own database as no single previously established database allowed for a comprehensive review of all injury types (including those admitted and discharged from the PED) and assessment of injury severity and complete demographics (including zip code data) in a timely manner. Children who present to non-pediatric-specific EDs were not included, and there may be variability in demographics, access to medical care, or safety practices in different regions of North America not captured in this study. Thus, there may be limitations to the generalizability of our findings. Nonetheless, this study represents a large sample size of pediatric patients presenting to PEDs with injuries with a wide geographic distribution to characterize injury patterns to children during the COVID-19 pandemic.

## Conclusion

The COVID-19 pandemic in 2020 is associated with increases in the proportion of children presenting to PEDs for injuries compared to 2019, including increases by specific injury mechanism. There were differences by race and payor among those seeking care for injuries. The changes in the distribution of injury types and severity seen in the PED should be factored into the preparation for response to any pandemic, inclusive of prevention efforts. The understanding of changes in injury patterns associated with the COVID-19 pandemic can serve as a catalyst for future injury prevention initiatives including state and national policy injury-specific policy changes and informing community and hospital partnerships.

## Data Availability

The datasets generated and/or analyzed during the current study are not publicly available due to limitations in the data use agreements with the 40 sites but are available from the corresponding author on reasonable request.

## Abbreviations

US: United States
PED: Pediatric Emergency Department
IFCK: Injury Free Coalition for Kids
ICD-10: International Classification of Disease-10th revision
MOO: Manual of Operations
ESI: Emergency Severity Index
ISS: Injury Severity Score
AIS: Abbreviated Injury Scale
CDC: Centers for Disease Control and Prevention
